# Design of a randomised controlled trial on immune effects of acidic and neutral oligosaccharides in the nutrition of preterm infants: carrot study

**DOI:** 10.1186/1471-2431-8-46

**Published:** 2008-10-23

**Authors:** Elisabeth AM Westerbeek, Ruurd M van Elburg, Anemone van den Berg, Jolice van den Berg, Jos WR Twisk, Willem PF Fetter, Harrie N Lafeber

**Affiliations:** 1 Department of Paediatrics, Division of Neonatology, VU University Medical Center, Amsterdam, The Netherlands; 2 Institute of Research in Extramural Medicine, VU University Medical Center, Amsterdam, The Netherlands

## Abstract

**Background:**

Prevention of serious infections in preterm infants is a challenge, since prematurity and low birth weight often requires many interventions and high utility of devices. Furthermore, the possibility to administer enteral nutrition is limited due to immaturity of the gastrointestinal tract in the presence of a developing immune system. In combination with delayed intestinal bacterial colonisation compared with term infants, this may increase the risk for serious infections. Acidic and neutral oligosaccharides play an important role in the development of the immune system, intestinal bacterial colonisation and functional integrity of the gut. This trial aims to determine the effect of enteral supplementation of acidic and neutral oligosaccharides on infectious morbidity (primary outcome), immune response to immunizations, feeding tolerance and short-term and long-term outcome in preterm infants. In addition, an attempt is made to elucidate the role of acidic and neutral oligosaccharides in postnatal modulation of the immune response and postnatal adaptation of the gut.

**Methods/Design:**

In a double-blind placebo controlled randomised trial, 120 preterm infants (gestational age <32 weeks and/or birth weight <1500 gram) are randomly allocated to receive enteral acidic and neutral oligosaccharides supplementation (20%/80%) or placebo supplementation (maltodextrin) between day 3 and 30 of life. Primary outcome is infectious morbidity (defined as the incidence of serious infections). The role of acidic and neutral oligosaccharides in modulation of the immune response is investigated by determining the immune response to DTaP-IPV-Hib(-HBV)+PCV7 immunizations, plasma cytokine concentrations, faecal Calprotectin and IL-8. The effect of enteral acidic and neutral oligosaccharides supplementation on postnatal adaptation of the gut is investigated by measuring feeding tolerance, intestinal permeability, intestinal viscosity, and determining intestinal microflora. Furthermore, short-term and long-term outcome are evaluated.

**Discussion:**

Especially preterm infants, who are at increased risk for serious infections, may benefit from supplementation of prebiotics. Most studies with prebiotics only focus on the colonisation of the intestinal microflora. However, the pathways how prebiotics may influence the immune system are not yet fully understood. Studying the immune modulatory effects is complex because of the multicausal risk of infections in preterm infants. The combination of neutral oligosaccharides with acidic oligosaccharides may have an increased beneficial effect on the immune system. Increased insight in the effects of prebiotics on the developing immune system may help to decrease the (infectious) morbidity and mortality in preterm infants.

**Trial registration:**

Current Controlled Trials ISRCTN16211826.

## Background

Preterm infants are at increased risk for the development of serious nosocomial infections, especially very low birth weight infants at a NICU [1]. In a recent review of the literature, we found that the intestinal bacterial colonisation in preterm infants is much more diverse than in term infants and that antibiotics cause a significant delay in the intestinal bacterial colonisation [2]. Furthermore, the possibility to administer enteral nutrition is limited due to immaturity of the gastrointestinal tract in the presence of a developing immune system.

Human milk has anti-inflammatory effects and bifidogenic effects on the intestinal microflora [3,4]. Term breastfed infants have less infections and develop less atopy compared with formula fed infants [5,6]. Many factors have been implicated in this effect, including human milk oligosaccharides [7,8]. Many attempts have been made to mimic this effect of human milk. Addition of prebiotics, consisting of neutral oligosaccharides, to infant formula has been found to show potential advantageous effects in term and preterm infants [9,10]. Besides neutral oligosaccharides, breast milk also contains acidic oligosaccharides [8]. In the past, research has mainly focussed on neutral oligosaccharides such as galacto-oligosaccharides and fructo-oligosaccharides (GOS/FOS). Supplementation of GOS/FOS in term and preterm infants results in: 1. Stimulation of a bifidogenic intestinal flora [11,12]; 2. Reduction of pathogens in the intestine [12]; 3. Production of beneficial fermentation metabolites such as short chain fatty acids (SCFA) [10]; 4. Decrease of stool pH [13]; 5. Improved intestinal physiology (stool characteristics, motility) [14]; 6. Less infections and atopy [15,16].

In breast milk 80% of the oligosaccharides are neutral (as in GOS/FOS), and 20% are acidic. Acidic oligosaccharides (AOS) can be derived from carrots with their active component pectin. Pectin is a common structural component of all higher plants. Cooking of pectin-containing vegetables induces the cleavage of the long-chain pectin polymers into acidic oligosaccharides. For already nearly 100 years, carrots are known to have health promoting effects. In 1908, carrot soup was used as treatment of diarrhoea [17]. In 1997, Guggenbichler identified the anti-adhesive effect of acidic oligosaccharides [18].

The combination of acidic and neutral oligosaccharides may have several advantageous effects [10,19-21]: 1. Improvement of the response to immunizations; 2. Stimulation of Th1 cytokine response (e.g. TNF-α, IFN-gamma) and decreasing the Th2 cytokine release (e.g. IL-10, IL-4, IL-5); 3. Stimulation of a bifidogenic intestinal flora; 4. Preventing adhesion of pathogens to epithelial tissues.

As a result of these effects, we hypothesise that preterm infants receiving a combination of GOS/FOS with AOS may have: 1. Less infections; 2. Better response to immunizations; 3. Less atopy later in life; 4. Less feeding intolerance.

As infections are still a major cause of morbidity and mortality in preterm infants, reducing the incidence of serious infections is very important. Controversy exists on the definitions for serious infections in neonates. Therefore in a previous study, we adjusted the criteria of the Centers for Disease Control and Prevention for serious infections in children < 1 year for use in neonates [1], and found in a prospective study these criteria applicable in preterm infants [22].

In conclusion, this double-blind randomised controlled trial aims to determine the effect of enteral supplementation of acidic and neutral oligosaccharides on infectious morbidity (primary outcome), immune response to immunizations, feeding tolerance and short-term and long-term outcome in preterm infants. In addition, an attempt is made to elucidate the role of acidic and neutral oligosaccharides in postnatal modulation of the immune response and postnatal adaptation of the gut.

## Methods/Design

The study is designed as a double-blind placebo controlled randomised clinical trial. Approval of the study protocol by the medical ethical review board of VU University Medical Center Amsterdam is obtained before the start of the study.

### Study population

Infants with a gestational age <32 weeks and/or birth weight <1500 gram admitted to the level III neonatal intensive care unit (NICU) of the VU University Medical Center, Amsterdam, are eligible for participation in the study. Written informed consent is obtained from all parents.

Exclusion criteria are: major congenital or chromosomal anomalies, death <48 hours after birth, transfer to another hospital <48 hours after birth and admission from an extra regional hospital.

### Treatment allocation and blinding

To balance birth weight distribution into treatment groups, each infant is stratified to one of three birth weight groups (≤ 799 g, 800–1199 g, ≥ 1200 g) and randomly allocated to treatment within 48 hours after birth. An independent researcher uses a computer-generated randomisation table (provided by Danone Research, Friedrichsdorf, Germany) to assign infants to treatment N or O. Investigators, parents, medical and nursing staff are unaware of treatment allocation. The randomisation code is broken after data analysis is performed.

### Treatment

Acidic and neutral oligosaccharides powder and the placebo powder (maltodextrin) are prepared by Danone Research, Friedrichsdorf, Germany and are packed sterile. During the study period, acidic and neutral oligosaccharides and placebo powder are monitored for stability and microbiological contamination.

Between days 3 and 30 of life, acidic and neutral oligosaccharides supplementation (20%/80% mixture) is administered in a dose of maximal 1.5 g/kg/day to breast milk or preterm formula in the intervention group. Two members of the nursing staff daily add supplementation to breast milk or to preterm formula (Nenatal Start^®^, Nutricia Nederland B.V., Zoetermeer, The Netherlands), according to the parents' choice. Per 100 mL, Nenatal Start^® ^provides 80 kcal, 2.4 g protein (casein-whey protein ratio 40:60), 4.4 g fat, and 7.8 g carbohydrate. When infants are transferred to another hospital before the end of the study, the protocol is continued under supervision of the principal investigator (EW).

### Nutritional support

Protocol guidelines for the introduction of parenteral and enteral nutrition follow current practice at our NICU. Nutritional support is administered as previously described [23].

For each infant in the study, a feeding schedule is proposed based on birth weight and the guidelines as mentioned above. However, the medical staff of our NICU has final responsibility for the administration of parenteral nutrition and advancement of enteral nutrition.

After discharge all infants receive breast milk or preterm formula Nenatal Start (without GOS/FOS)^® ^until term, and Nenatal 1 (without GOS/FOS)^® ^until the corrected age of 6 months.

### Study outcome measures

#### Clinical outcome measures

Primary outcome of the study is the effect of acidic and neutral oligosaccharides (20%/80% mixture) supplemented to the enteral nutrition on infectious morbidity as previously defined [1,22]. The occurrence of serious infections is determined by two investigators, unaware of treatment allocation, as previously described [1,22].

The following perinatal characteristics are registered to assess prognostic similarity: maternal age and race, obstetric diagnosis, administration of antenatal steroids and antibiotics, mode of delivery, sex, gestational age, birth weight, birth weight <10^th ^percentile [24], Apgar scores, pH of the umbilical artery, clinical risk index for babies [25], and administration of surfactant.

During the study period, actual intake of enteral and parenteral nutrition, powder supplementation and type of feeding (breast milk or preterm formula) are recorded daily. Feeding tolerance and short-term outcome are evaluated. (Table 1)

**Table 1:** Clinical outcome measures

	**Remarks**
	
**Infectious morbidity**	
Serious infections	Primary outcome
Number of infectious episodes	
Cultured micro-organisms	
	
**Feeding tolerance**	
Enteral feeding >120 mL/kg/day	
Age at finishing parenteral nutrition	
Necrotising enterocolitis	Bell et al.[35]
	
**Short-term outcome**	
Weight *z *scores at birth, day 30 and at discharge	Usher et al. [24]
Patent ductus arteriosus	
Ventilatory support	
Use of oxygen at postmenstrual age of 36 weeks	Jobe et al. [36]
Intraventricular hemorrhage	Papile et al. [37]
Retinopathy of prematurity	Committee for ROP. [38]
Death	
Age at discharge from NICU and age at discharge home	

ROP = retinopathy of prematurity; NICU = neonatal intensive care unite.

#### Immune response

The effect of acidic and neutral oligosaccharides supplemented enteral nutrition on the immune response is investigated, in collaboration with the National Institute for Public Health and Environment, by determining the development of the immune response to DTaP-IPV-Hib(-HBV) + PCV7 immunizations (after the first 3 doses), and the development of the memory function of the immune response to these immunizations by measuring the response after the 4^th ^booster dose. In addition, the plasma cytokine concentrations (Il-2, Il-4, IL-5, Il-8, IL-10, TGF, IFN), faecal Calprotectin measured by ELISA (Buhlmann, Switzerland), and IL8 measured by random-access chemiluminescence immunoassay (Siemens, The Netherlands) are determined.

#### Postnatal adaptation of the gut

The effect of acidic and neutral oligosaccharides supplemented enteral nutrition on postnatal adaptation of the gut is studied by measuring feeding tolerance, intestinal permeability, intestinal microflora and intestinal viscosity.

Intestinal permeability is measured by the sugar absorption test [26]. After instillation of the test solution, 2 mL/kg by nasogastric tube, urine is collected for 6 hours. After collection, 0.1 mL chlorohexidine digluconate 20% (preservative) is added to the urine and samples are stored at -20°C until analysis. Lactulose and mannitol concentrations (mmol/mol creatinine) are measured by gas chromatography as previously described [27]. The lactulose/mannitol ratio is calculated and used as a measure of intestinal permeability.

Faecal samples are stored at -20°C until analysis by fluorescent in situ hybridisation (FISH) using specific 16S rDNA-targeted [28]. Intestinal viscosity is measured by high-pressure capillary rheometry (viscosimetry) as described by Mihatsch et al. [14].

#### Long-term outcome

To determine the incidence of allergic and infectious disease in the first year of life standardized questionnaires will be sent to the parents prior to the follow-up visit at the corrected age of 1 year [29]. Faecal samples (FISH, Calprotectin and IL-8) and IgE/IgG4 levels in blood will be measured at the age of 5 and 12 months.

To investigate neurodevelopmental outcome, neurological status, vision, hearing and Mental Development Index (MDI) and Psychomotor Development Index (PDI) of the Bayley Scales of Infant Development II (BSID-II) at the corrected age of 1 and 2 years (as part of the regular follow-up of NICU infants) are assessed [30,31].

To determine the frequency of side-effects after the first 4 immunizations, standardized questionnaires will be given to the parents at the time of immunizations. (Table 2)

**Table 2:** Study Schedule

	**< 48 h**	**Day 4**	**day 7**	**day 14**	**day 30**	**5 months**	**1 year**	**2 year**
**Immune response**								
Response to immunizations						x	x	
Cytokine response	x		x	x		x	x	
Faecal Calprotectin/IL-8	x		x	x	x	x	x	

**Postnatal adaptation of the gut**								
Intestinal permeability	x	x	x					
Intestinal microflora	x		x	x	x	x	x	
Intestinal viscosity					x	x	x	

**Long-term outcome**								
IgE/IgG4	x					x	x	
Allergic and infectious diseases							x	
Side effects immunizations^†^								
Neurodevelopment							x	x

^†^Standardized questionnaires (Preparedness and Response Unit, Centre for Infectious disease Control Netherlands, National Institute for Public Health and the Environment, The Netherlands) after the 1^st^, 2^nd^, 3^rd ^and 4^th ^immunization.

### Sample size

Based on the differences in incidences in infectious morbidity (76% and 50% respectively) in the GEEF study [22], and a two-tailed α = 0.05, β = 0.20, a sample size of 2 × [2*7.85*0.63(0,37)]/(0,26)^2 ^= 2 × 54 infants is calculated. Based on an expected drop-out rate of 10% during the study period 2 × 60 infants will be included.

### Statistical analysis

To determine whether randomisation is successful, prognostic similarity (perinatal and nutritional characteristics) between treatment groups is assessed. The Students' t-test, Mann-Whitney U test, and chi-square test or Fisher's exact test are used to compare continuous normally distributed, nonparametric continuous and dichotomous data respectively.

Logistic regression is performed to examine whether acidic and neutral oligosaccharide supplemented enteral nutrition decreases the incidence of serious infections. In an additional analysis, adjustments are made for possible confounding factors such as administration of antenatal corticosteroids, birth weight <10th percentile and administration of breast milk. Analyses of secondary outcomes (only crude) is performed by Students' t- test, Mann-Whitney U test, chi-square test or Fisher's exact test for (non)parametric continuous, dichotomous data and time-dependent data respectively.

Generalised estimated equations [32] are used to analyse differences and changes over time in plasma cytokine concentrations, faecal Calprotectin and IL-8, intestinal permeability, intestinal microflora and intestinal viscosity. Differences of optimal and non-optimal neuromotor development and normal and abnormal mental/motor development in oligosaccharides and control groups is examined by logistic regression with adjustments for possible confounding factors as gestational age and birth weight.

All statistical analyses are performed on an intention to treat basis. In addition, alternative per protocol analyses are performed, excluding all patients who are not treated according to protocol, defined as more than 3 consecutive days or a total of 5 days on minimal enteral feeding or without supplementation. For all statistic analyses a p value <0.05 is considered significant (two-tailed). SPSS 15.0 (SPSS Inc., Chicago, IL, USA) is used for data analysis.

## Discussion

There is increasing evidence that prebiotics play an important role in the development of the intestinal microflora and the immune system, and may help to decrease the risk of infectious diseases. Especially preterm infants, who are at increased risk for serious infections, may benefit from supplementation of prebiotics. Most studies with prebiotics only focus on the colonisation of the intestinal microflora. The influence on the immune system is not yet fully understood [33]. Studying the immune modulatory effects is complex because of the multicausal risk of infections in preterm infants [34]. The combination of neutral oligosaccharides with acidic oligosaccharides may have an increased beneficial effect on the immune system of preterm infants due to the specific conditions in the luminal part of the developing gut wall. Not only the immune effects, such as morbidity due to infections and response to immunizations will be investigated, but also other signs and symptoms such as feeding tolerance, short-term, long-term and postnatal adaptation of the gut (intestinal microflora, intestinal permeability, intestinal viscosity). Increased insight in the effects of prebiotics on the developing immune system may help to find ways to decrease the (infectious) morbidity and mortality in preterm infants.

## Competing interests

Danone Research (Friedrichsdorf, Germany) for the financial support and for providing Nenatal Start^®^, Nenatal 1^®^, acidic and neutral oligosaccharides and placebo supplementation.

## Authors' contributions

Ruurd M van Elburg, Elisabeth AM Westerbeek and Harrie N Lafeber formulated the research question and wrote the study protocol. Anemone van den Berg, Willem P Fetter and Jolice van den Berg contributed to the development of the protocol. Jos WR Twisk gave advice on statistical analysis. Elisabeth AM Westerbeek and Ruurd M van Elburg wrote the draft for this manuscript and the other authors reviewed the manuscript. All authors approved the final version of the manuscript.

## Pre-publication history

The pre-publication history for this paper can be accessed here:


